# Supplementary material for: Rad1 and Rad10 Tied to Photolyase Regulators Protect Insecticidal Fungal Cells from Solar UV Damage by Photoreactivation
Source: J Fungi (Basel). 2022 Oct 25;8(11):1124. doi: 10.3390/jof8111124 (PMC9692854; doi:10.3390/jof8111124)
Supplement: Supplementary file 1 [file jof-08-01124-s001.zip › jof-1979747-supplementary.pdf]

Supplementary Material for Journal of Fungi

Rad1 and Rad10 Tied to Photolyase Regulators Protect Insecticidal Fungal Cells from Solar UV Damage by Photoreactivation

Lei Yu†, Si-Yuan Xu†, Xin-cheng Luo, Sheng-Hua Ying, Ming-Guang Feng*

Institute of Microbiology, College of Life Sciences, Zhejiang University, Hangzhou, Zhejiang, 310058, China

† These authors contribute equally to this study.

* For correspondence. E-mail: mgfeng@zju.edu.cn (M.G.F); Tel/Fax: (+)86 571 88206178

Table S1. Paired primers used for manipulation and detection of target genes in *B. bassiana*.

Primers	Paired sequences (5′–3′)	Purpose
cRad1-F/R	CAATCACAAACACCTTCAAAATGTCGACAAACAATGCGCC / TCCTCGCCCTTGCTCAC<br>CATGTAATCTTCTTCGTCCATGA	Cloning *rad1* cDNA (2820 bp) for fusion to *gfp*
cRad10-F/R	CAATCACAAACACCTTCAAAATGGCTGACGAATACGGCGC / TCCTCGCCCTTGCTCA<br>CCATTTTGCGAAGCTTTGCCAGCG	Cloning *rad10* cDNA (1176 bp) for fusion to *gfp* or *mCherry*
adRad1-F/R	GCCATGGAGGCCAGT**GAATTC**ATGTCGACAAACAATGCGCC / CAGCTCGAGCTCGAT<br>**GGATCC**GTAATCTTCTTCGTCCATGA	Cloning *rad1* cDNA (2820 bp) for ligation to AD (*EcoR*I/*BamH*I)
bdRad1-F/R	ATGGCCATGGAGGCC**GAATTC**ATGTCGACAAACAATGCGCC / CGCTGCAGGTCGAC<br>**GGATCC**GTAATCTTCTTCGTCCATGA	Cloning *rad1* cDNA (2820 bp) for ligation to BD (*EcoR*I/*BamH*I)
adRad10-F/R	GCCATGGAGGCCAGT**GAATTC**ATGGCTGACGAATACGGCGC / CAGCTCGAGCTCGAT<br>**GGATCC**TTTGCGAAGCTTTGCCAGCG	Cloning *rad10* cDNA (1176 bp) for ligation to AD (*EcoR*I/*BamH*I)
bdRad10-F/R	ATGGCCATGGAGGCC**GAATTC**ATGGCTGACGAATACGGCGC / CGCTGCAGGTCGAC<br>**GGATCC**TTTGCGAAGCTTTGCCAGCG	Cloning *rad10* cDNA (1176 bp) for ligation to BD (*EcoR*I/*BamH*I)
adWC1-F/R	GCCATGGAGGCCAGT**GAATTC**ATGGAAGGATACTACCCTCC / CAGCTCGAGCTCGAT<br>**GGATCC**AGGTAAGCTCGTTTCACGCT	Cloning *wc1* cDNA (2889 bp) for ligation to AD (*EcoR*I/*BamH*I)
adWC2-F/R	GCCATGGAGGCCAGT**GAATTC**ATGTCCCAGGGACACGCGCC / CAGCTCGAGCTCGAT<br>**GGATCC**GCTTCCGGCACCAAACTCTG	Cloning *wc2* cDNA (1497 bp) for ligation to AD (*EcoR*I/*BamH*I)
bdWC1-F/R	ATGGCCATGGAGGCC**GAATTC**ATGGAAGGATACTACCCTCC / CGCTGCAGGTCGAC<br>**GGATCC**AGGTAAGCTCGTTTCACGCT	Cloning *wc1* cDNA (2889 bp) for ligation to BD (*EcoR*I/*BamH*I)
bdWC2-F/R	ATGGCCATGGAGGCC**GAATTC**ATGTCCCAGGGACACGCGCC / CGCTGCAGGTCGAC<br>**GGATCC**GCTTCCGGCACCAAACTCTG	Cloning *wc2* cDNA (1497 bp) for ligation to BD (*EcoR*I/*BamH*I)
adPhr1-F/R	GCCATGGAGGCCAGT**GAATTC**ATGGCGCCTCGTGCAACGAA / CAGCTCGAGCTCGAT<br>**GGATCC** CGCCACAGCCGCCTTGTACG	Cloning *phr1* cDNA (1761 bp) for ligation to AD (*EcoR*I/*BamH*I)
bdPhr1-F/R	ATGGCCATGGAGGCC**GAATTC**ATGGCGCCTCGTGCAACGAA / CGCTGCAGGTCGAC<br>**GGATCC**CGCCACAGCCGCCTTGTACG	Cloning *phr1* cDNA (1761 bp) for ligation to BD (*EcoR*I/*BamH*I)
adPhr2-F/R	GCCATGGAGGCCAGT**GAATTC**ATGACAAAGCCCAGAGTCAT / CAGCTCGAGCTCGAT<br>**GGATCC**CGTTTTCTGCTTCTTCGCTG	Cloning *phr2* cDNA (1869 bp) for ligation to AD (*EcoR*I/*BamH*I)
bdPhr2-F/R	ATGGCCATGGAGGCC**GAATTC**ATGACAAAGCCCAGAGTCAT / CGCTGCAGGTCGAC<br>**GGATCC**CGTTTTCTGCTTCTTCGCTG	Cloning *phr2* cDNA (1869 bp) for ligation to BD (*EcoR*I/*BamH*I)
Pphr1-F/R	AAGCTTGAATTCGAGCTCGGTATCCACGCCGTCGAATAAG / CTCGAGGTCGACAGATC<br>**CCCGGG**AAGGGTACGCGATGGTAATTA	Cloning P*phr1* (1829 bp) (*Xma*I)
Pphr2-F/R	AAGCTTGAATTCGAGCTCGGTAATACTGGGGACAGACACTTGC / CTCGAGGTCGACA<br>GATC**CCCGGG**GTTTCCTGATTCGAAACTGGA	Cloning P*phr2* (1904 bp) (*Xma*I)
upRad1-F/R	ACGAGCTGTACAAGTAA**CCCGGG**GGCGTTGGAGCCTGATT / TGGCTGCAGGTCGAC<br>**GGATCC**CGAATGCTTCAGCGTGTTT	Cloning *rad1* 5'-end (1593 bp) for disruption (*Xma*I/*BamH*I)
dnRad1-F/R	GACCCATGGCTCGAG**TCTAGA**GGTTGTAAACACCGACTTTCAGA / GGTGGTGGTGG<br>CTAGC**GTTAAC**GACGCACAGAGGCAAGGTAG	Cloning *rad1* 3'-end (1673 bp) for disruption (*Xba*I/*Hpa*I)
upRad10-F/R	ACGAGCTGTACAAGTAA**CCCGGG**GCTGTCGGAGACGAGGTA / CGGTACCAAGCTTG<br>G**CTGCAG**GAACGGATCGGAAGAAAG	Cloning *rad10* 5'-end (1633 bp) for disruption (*Xma*I/*PstI*)
dnRad10-F/R	GACCCATGGCTCGAG**TCTAGA**GTTCGCCGTCTAGTCTCAA / GGTGGTGGTGGCTAGC<br>**GTTAAC**TGCCCTCCTGTCTCGTTA	Cloning *rad10* 3'-end (1853 bp) for gene disruption (*Xba*I/*Hpa*I)
upWC1-F/R	AGCTGTACAAGTAA**CCCGGG**ACCATAGAAGGACCGAAGGC / TGGCTGCAGGTCGAC<br>**GGATCC**AGCCCTGATCCTCTTCTCAAA	Cloning *wc1* 5'-end (1007 bp) for t disruption (*Xma*I/*BamH*I)
dnWC1-F/R	GACCCATGGCTCGAG**TCTAGA**GCGCACGACATCCGATAC / GGTGGTGGTGGCTAGC<br>**GTTAAC**CTCTGCCGACCCGAACA	Cloning *wc1* 3'-end (1136 bp) for disruption (*Xba*I/*Hpa*I)
upWC2-F/R	AGCTGTACAAGTAA**CCCGGG**GATGCTATTCCAGCGACTTTT / TGGCTGCAGGTCGAC<br>**GGATCC**GAGCGAGTCGTGTCTACCG	Cloning *wc2* 5'-end (1190 bp) for disruption (*Xma*I/*BamH*I)
dnWC-F/R	GACCCATGGCTCGAG**TCTAGA**CGCTGGGCGAAGAAGGA / GGTGGTGGTGGCTAGC<br>**GTTAAC**CGACGCACTGTTTGGGAAG	Cloning *wc2* 3'-end (723 bp) for disruption (*Xba*I/*Hpa*I)
flRad1-F/R	ATCCGTCGACCTGCAGCC**AAGCTT**GGGGAGCAACCACAAAGT / ACACTAGTCAGAT<br>CTTC**TCTAGA**CCAGGATAGGAATCGCAAA	Cloning full-length *rad1* (6680 bp) for complementation (*Hind*III/*Xba*I*)*
flRad10-F/R	ATCCGTCGACCTGCAGCC**AAGCTT**TTGCCTTCGTCGTAACCC / ACACTAGTCAGATCT<br>TC**TCTAGA**AATGCCCTCCTGTCTCGT	Cloning full-length *rad10* (4371 bp) for complementation (*Hind*III/*Xba*I*)*
flWC1-F/R	ATCCGTCGACCTGCAGCC**AAGCTT**CTCAACGCCGTAGTCCCATA/ACACTAGTCAGATCT<br>TC**TCTAGA**GCGGTCGTGAGGTGATTGT	Cloning full-length *wc1* (6362 bp) for complementation (*Hind*III/*Xba*I*)*
flWC2-F/R	ATCCGTCGACCTGCAGCC**AAGCTT**CCATAGAAGGACCGAAGGCT / ACACTAGTCAGAT<br>CTTC**TCTAGA**AGCGGTCGTGAGGTGATTG	Cloning full-length *wc2* (4790 bp) for complementation (*Hind*III/*Xba*I*)*
pRad1-F/R	TGAAACCCATCTCAGCACAC / CACCAGCGGCATACATCTT	PCR detecting *rad1*
pRad10-F/R	AATCGTCGTCACGGAACTT / GGATGAAGGCGGTGGTATT	PCR detecting *rad10*
pWC1-F/R	CGCTACCCGAATACTAACCAAT / ACAAACGCCCTATGACGGA	PCR detecting *wc1*
pWC2-F/R	CGCTTATTGTCATCATCTGTTCC / TGGTACGCTTCTTCTCCTTCTTC	PCR detecting *wc2*

* The underlined regions are DNA fragments to exchange for the corresponding fragments of constructed vectors at the sites(in bold) of indicated restriction enzymes.

Table S2. Paired primers used for the qPCR analyses of target genes in *B. bassiana*.

Gene	Tag locus[a]	Annotation[b]	Sequences (5′–3′) of paired primers
rad1	BBA_07749	Rad1 ortholog	TCGTCACCAACCTACTGCAC / CGGCATACATCTTTTCGCGG
rad10	BBA_03417	Rad10 ortholog	TCAGGATCAGGAGGCAAAGT / CAGCAAGCATGTCGTTGTTC
wc1	BBA_10271	White collar 1	GACCATGCAATTCAACAACG / GTATCGCTTGATCGACAGCA
wc2	BBA_01403	White collar 2	CCAGTCTCCTTTCTGCCAAG / TGGCAGATGAACCAGTCAAG
phr1	BBA_01664	CPD photolyase	ACTCATAGACTGGCGCATGG / TTTTCGCCTTGTCTCCAGCA
phr2	BBA_01034	6-4 PP photolyase	CACAGGCAAGACGTACCCC / CGTCGTCACTCTCCAGAACA
rad2	BBA_00274	Rad2 ortholog	ATGGGCGTGAACGGTCTTT / ATCAAAGACGAAGACGGGCT
rad3A	BBA_10031	Rad3 homolog 1	AAGGCAAGGCTGGAATTTTT / CAAGCCCTGATTGATCCACT
rad3B	BBA_00658	Rad3 homolog 2	ATGGTGAAGAGGGAATCACG / CAGGCCTTCTGGTAGAGACG
rad4A	BBA_02814	Rad4 homolog 1	AGTCCGATATGCAAAGGCGT / TACTTCGTGGCGCCGTAAAT
rad4B	BBA_02963	Rad4 homolog 2	CCTGATGGTACAGCCAAGGA/CGTGCTCTCTTCCACTTCGT
rad14	BBA_03454	Rad14 ortholog	CTGCTTCCCCATCTATCCAA / TCCGTCAGCTTCTCCCTAAA
rad16A	BBA_07794	Rad16 homolog 1	TTCGCAGTTTACAACGATGC / AGGAAACACTCCACGTCCAC
rad16B	BBA_01511	Rad16 homolog 2	CTCTAGTTCCGTCGCCAAAG / AACGCAGGTTGCTATCGAGT
rad16C	BBA_03842	Rad16 homolog 3	GGCTCCAATGTCCTTGTTGT / TGACTCCGTAGCTCGTGATG
rad23	BBA_01030	Rad23 ortholog	AGCAGAAATTCACCCTCGAA / CAGACAACGAAGCCCTTTTC
rad25	BBA_01121	Rad25 ortholog	CACATGCCTTCGAGATTGCG / TTGCCTCGTCATGTCGGAAA
rfa1	BBA_04757	RFA1 ortholog	ATGGTTGACGATACGGGGTA / GAGTCGTACCACCCCTTCAA
rfa2	BBA_05125	RFA2 ortholog	CTTCGGTGGTCACGGTATCT / TCGTCAAATCATCCCAGTCA
tfb1	BBA_08089	TFIIH subunit TFB1	AATCATGTCCCAGCAAGTCC / ACTCAGCTCTGGCGTCATTT
tfb2	BBA_05749	TFIIH subunit TFB2	GTGATGCGCATGCTTTACAT / TGCACTTCTTGCGTCTTGTC
tfb4	BBA_09804	TFIIH subunit TFB4	AAGGCCTACTCGCCTACCTC / GAGAGGCAAATGGAGCAGAC
tfb5	BBA_04362	TFIIH subunit TFB5	ATGCCTCGTGCCATAAGAAG / GGCAAGTTTTCCTTGAGACG
ssl1	BBA_08550	TFIIH subunit SSL1	GCAGGGGAACCCTAGCTTAC / GGATTCGGTCGCTGATAAGA
act	BBA_04860	β-actin	GGCAACATTGTCATGTCTGG / TTTGCTGGAAGGTGGATAGG

[a] Gene accession codes in *B. bassiana* genome under the NCBI accession NL_ADAH00000000.

[b] All listed genes except *wc1*, *wc2*, *phr1* and *phr2* are annotated as components of putative NER pathway by referring to Boiteux and Jinks-Robertson (2013)

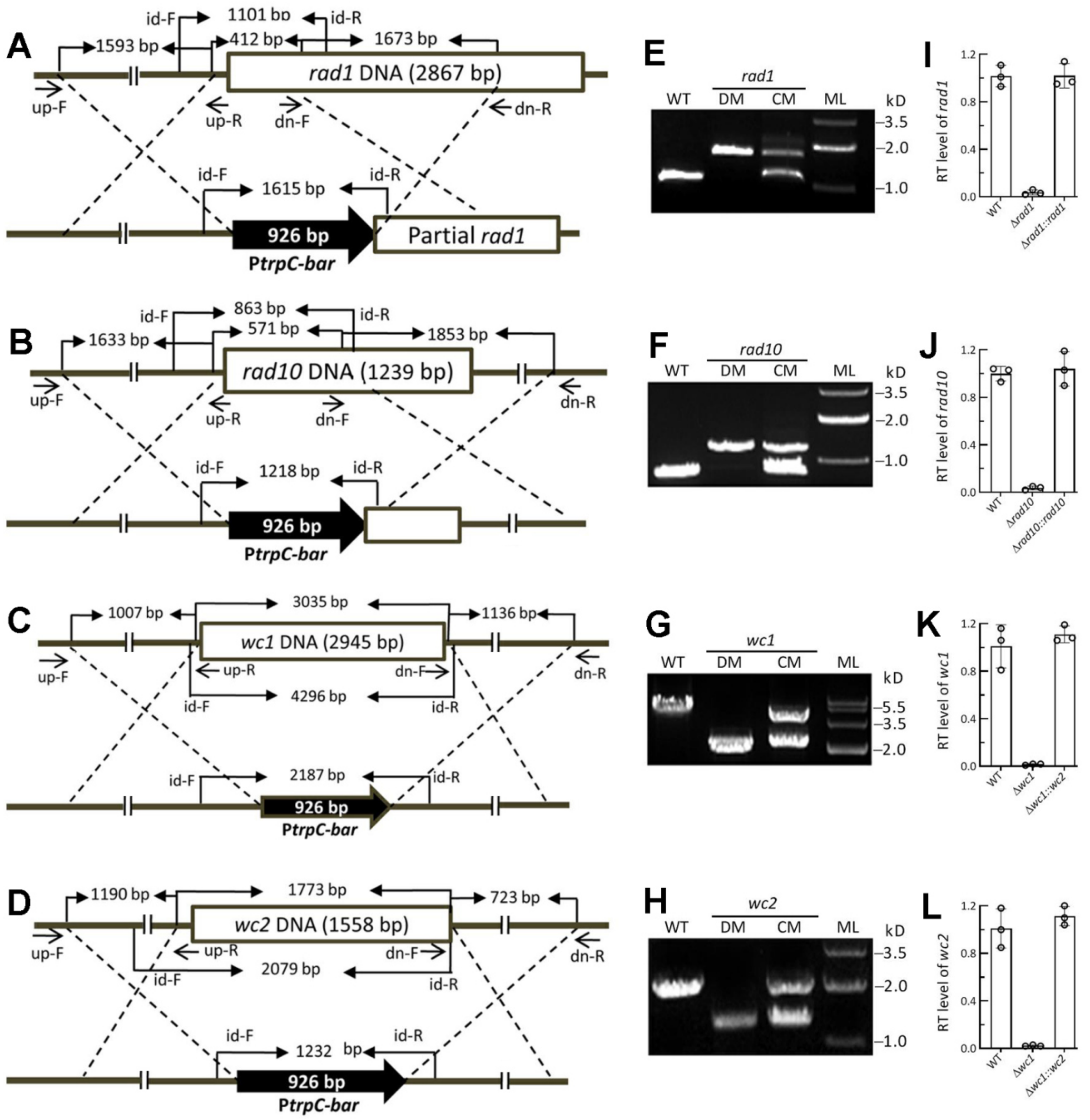

Figure S1. Generation and identification of *rad1, rad10, wc1* and *wc2* mutants in *B. bassiana*. (**A–D**) Schematic diagrams for targeted single-gene deletion strategies. (**E–H**) The *rad1, rad10, wc1* and *wc2* mutants identified via PCR analysis with paired primers (see Table S1), respectively. The detected DNA fragments indicate a success in deleting the partial or full-length coding and partial flanking regions of each target gene from the WT strain as expected and also in complementing it into the deletion mutant (1101 + 926 – 1615 = 412 bp for *rad1*, 863 + 926 – 1218 = 571 bp for *rad10*, 4296+ 926 – 2187 = 3035 bp for *wc1*, and 2079 + 926 – 1232 = 1773 bp for *wc2*). DM, deletion mutant. CM, complementation mutant. ML, molecular ladder of genomic DNA. (**I–L**) Relative transcript (RT) levels of *rad1, rad10, wc1* and *wc2* in the 3 d-old SDAY cultures of their deletion and complementation mutants with respect to the WT standard. Note that the expression of each target gene was not detectable in its DM but well restored in its CM. Error bars: standard deviations of the means from three cDNA samples derived from independent cultures of each strain.

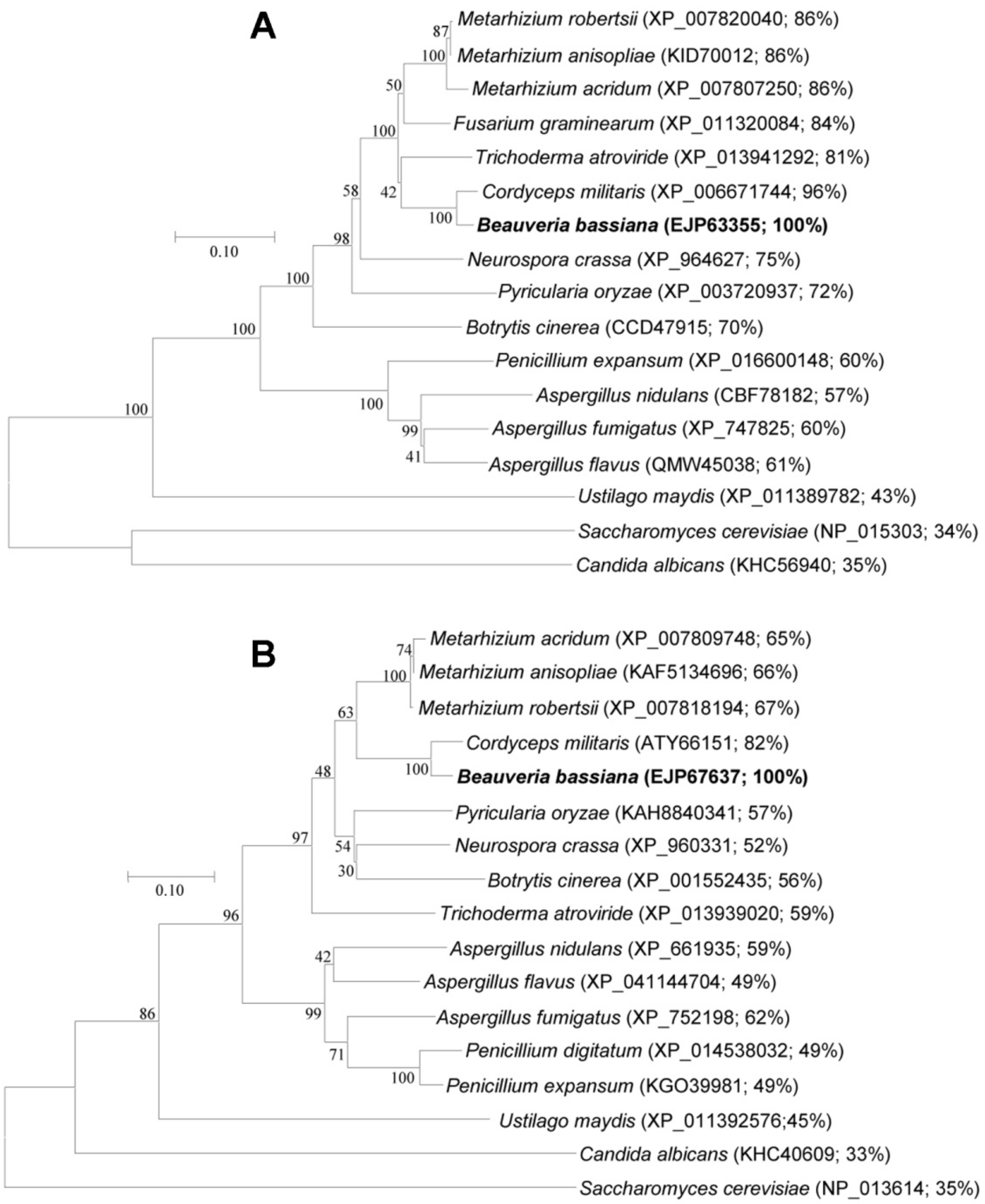

Figure S2. Phylogenetic relationships of Rad1 (**A**) and Rad10 (**B**) orthologues found in selected filamentous fungi. Each tree was constructed with the maximum likelihood method in the online program MEGA7 (http://www.megasoftware.net/) Bootstrap values of 1000 replications are shown at nodes. Scale bar: branch length proportional to genetic distance. The NCBI accession code of each protein and its protein sequence identity to its *B. bassiana* orthologue (in bold) are given in the parentheses following the name of its host fungus.

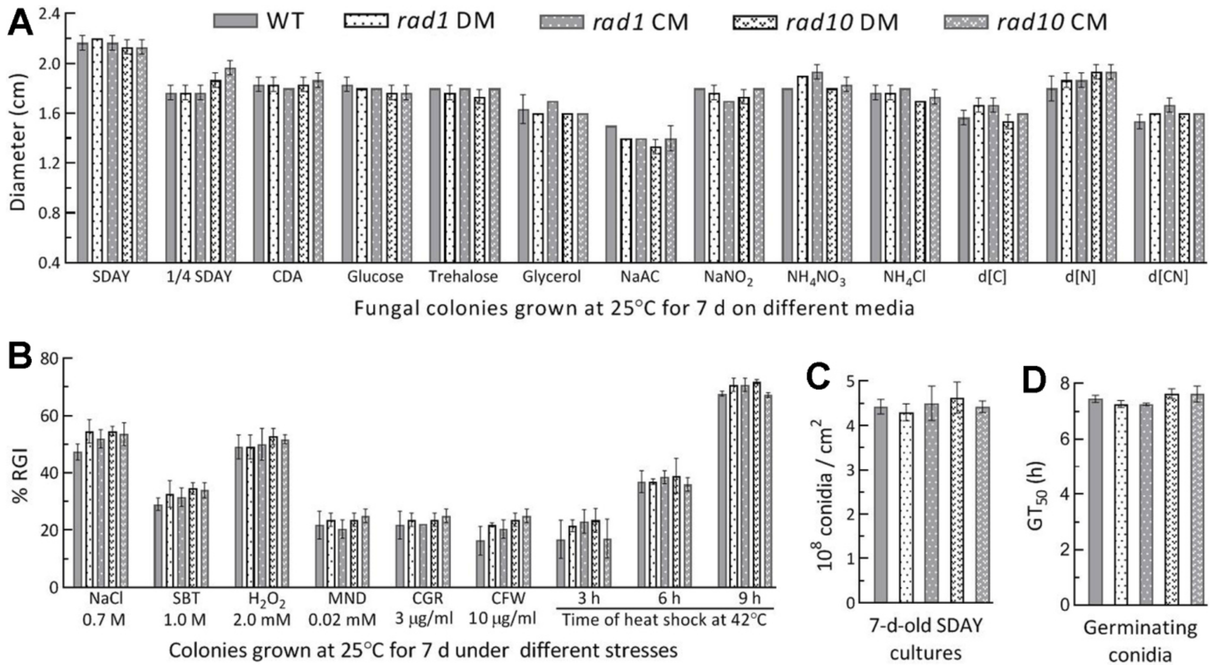

Figure S3. Dispensable roles of Rad1 and Rad10 in the asexual cycle *in vitro* of *B. bassiana*. (**A**) Diameters of fungal colonies (DM, deletion mutant; CM, complementation mutant) incubated at 25°C for 7 days on the plates of rich medium SDAY, 1/4 SDAY, minimal medium CDA and CDAs amended with different carbon or nitrogen sources and with the deletion of carbon (d[C]) or nitrogen (d[N]) source or both (d[CN]). (**B**) Relative growth inhibition (RGI) percentages of fungal colonies incubated at 25°C for 7 days on CDA plates supplemented with indicated concentrations of different chemical stressors (SBT, sorbitol; MND, menadione; CGR, Congo red; and CFW, calcofluor white) or for 5-day growth recovery after exposing 2-day-old SDAY colonies to a heat shock for 3, 6 and 9 h at 42°C. All fungal colonies were initiated by spotting 1 μL aliquots of a 10^6 conidia/mL suspension. (**C**) Conidial yields measured from the 7-day-old SDAY cultures initiated by spreading 100 μL aliquots of a 10^7 conidia/mL suspension at the optimal regime of 25°C and L:D 12:12. (**D**) Median germination time (GT_{50}) of conidia at 25°C. Error bars: standard deviations of the means from three independent replicates.

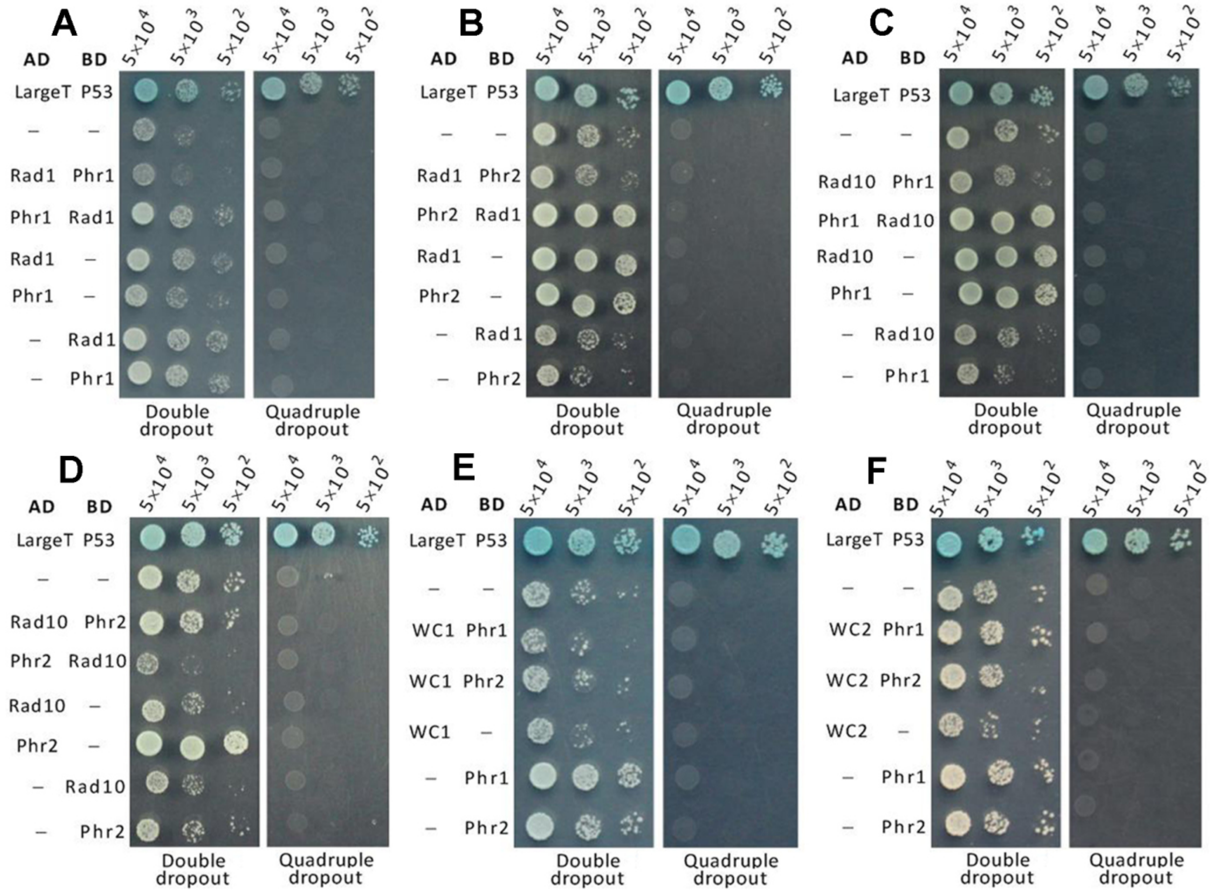

Figure S4. Y2H assays for protein-protein interactions in *B. bassiana*. Note no signals for interactions of either Rad1 or Rad10 with Phr1 and Phr2 (**A–D**) and of either WC1 (**E**) or WC2 (**F**) with Phr1 and Phr2.
